# Unconventional Breathing Currents Far beyond the Quantum Tunneling Distances in Large-Gapped Nanoplasmonic Systems

**DOI:** 10.1021/acs.nanolett.3c05133

**Published:** 2024-01-26

**Authors:** Aravind Satheesh, Chia-Ming Yang, Vilas Gaidhane, Neeru Sood, Nilesh Goel, Selim Bozkurt, Krishna Kumar Singh, Nikhil Bhalla

**Affiliations:** † Nanotechnology and Integrated Bioengineering Centre (NIBEC), School of Engineering, Ulster University, 2-24 York Street, Belfast BT15 1AP, Northern Ireland, United Kingdom; ‡ Department of Electronic Engineering, Chang Gung University, No. 259, Wenhua 1st Rd, Guishan District, Taoyuan City 33302, Taiwan (R.O.C.); § Institute of Electro-Optical Engineering, Chang Gung University, No. 259, Wenhua 1st Rd, Guishan District, Taoyuan City 33302, Taiwan (R.O.C.); ∥ Department of Neurosurgery, Chang Gung Memorial Hospital at Linkou, No. 5, Fuxing St, Guishan District, Taoyuan City 33305, Taiwan (R.O.C.); ⊥ Department of Materials Engineering, Ming Chi University of Technology, 84 Gungjuan Rd, Taishan District, New Taipei City 243303, Taiwan (R.O.C.); # Department of Electronic Engineering, Ming Chi University of Technology, 84 Gungjuan Rd, Taishan District, New Taipei City 243303, Taiwan (R.O.C.); ∇ Department of Electrical and Electronics Engineering, Birla Institute of Technology and Science (BITS), Pilani Dubai Campus, Dubai International Academic City, P.O. Box: 345055, Dubai, United Arab Emirates; ○ Department of Biotechnology, Birla Institute of Technology and Science (BITS), Pilani Dubai Campus, Dubai International Academic City, P.O. Box: 345055, Dubai, United Arab Emirates; ◆ Department of Physics, Birla Institute of Technology and Science (BITS), Pilani Dubai Campus, Dubai International Academic City, P.O. Box: 345055, Dubai, United Arab Emirates; ¶ Healthcare Technology Hub, Ulster University, 2-24 York Street, Belfast, BT15 1AP, Northern Ireland, United Kingdom; ▼ School of Engineering, Ulster University, Belfast, BT15 1AP, Northern Ireland, United Kingdom

**Keywords:** LSPR, Nanoplasmonics, Photonics, Nanoparticle circuit, Light-driven currents, Sensors

## Abstract

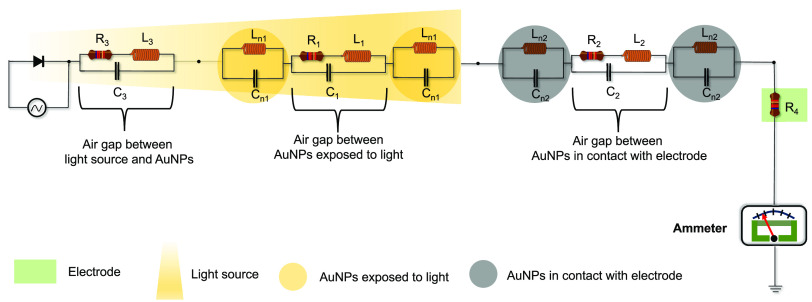

Localized surface plasmon resonance (LSPR) in plasmonic nanoparticles propels the field of plasmo-electronics, holding promise for transformative optoelectronic devices through efficient light-to-current conversion. Plasmonic excitations strongly influence the charge distribution within nanoparticles, giving rise to electromagnetic fields that can significantly impact the macroscopic charge flows within the nanoparticle housing material. In this study, we present evidence of ultralow, unconventional breathing currents resulting from dynamic irradiance interactions between widely separated nanoparticles, extending far beyond conventional electron (quantum) tunneling distances. We develop an electric analogue model and derive an empirical expression to elucidate the generation of these unconventional breathing currents in cascaded nanoplasmonic systems under irradiance modulation. This technique and theoretical model have significant potential for applications requiring a deeper understanding of current dynamics, particularly on large nanostructured surfaces relevant to photocatalysis, energy harvesting, sensing, imaging, and the development of future photonic devices.

The fundamental process of light-to-current conversion, achieved through the complex interplay of localized surface plasmon resonance (LSPR) excitations and their subsequent contribution to local electric field enhancements, stands as a pivotal catalyst propelling the rising realm of plasmo-electronics.^1−3^ This growing discipline holds the promise of giving birth to a revolutionary cohort of optoelectronic devices that is poised to transcend current materials science.^4,5^ In the past, optical field enhancement through LSPR active materials has led to enhanced Raman scattering spectroscopy,^6^ enabled nonlinear processes such as frequency mixing^7^ and single molecule bio/chemical sensing,^8^ and allowed for the enhancement of chemical reactions involving energy generation.^9^ Recent works have also unveiled the capacity of LSPR excitations to influence the charge flows on a macroscopic scale.^10−12^ In this context, the charging of metal nanoparticles (NPs) emerges as a potent modulator of charge flow, capable of shaping the plasmonic responsiveness of the NPs.^13^ Notably, the wave-like charge fluctuations observed in plasmonic nanoparticle systems have presented a compelling scenario for electrical current across large interparticle gaps, defying conventional expectations for thermalizd electrons.^14^ This phenomenon is attributed to the intense electromagnetic near-fields confined within inter-nanoparticle regions, bolstering electron movement and thereby amplifying current within large-scale assemblies of NPs.^15^

On the other hand, it is well-known that the intensity of incident light or the irradiance on the LSPR active material, which is used to generate LSPR, is a critical parameter that can be tuned to control and manipulate the behavior of LSPR.^16^ For instance, higher light intensities can generate “hot carriers”, which are essentially high-energy electrons and holes generated through LSPR.^17^ These hot carriers can contribute to various photochemical and photophysical processes, potentially influencing the overall plasmonic response of the NPs.^18,19^ Furthermore, the intensity of the light on LSPR NPs can lead to localized heating of the NPs, which can affect their plasmonic behavior.^20^ This thermal component can lead to changes in the dielectric properties of the surroundings of the NPs, affecting the shape and frequency of the LSPR spectrum.^21^ Therefore, the impact of intensity modulation can cascade across a panorama of applications spanning the breadth of sensing, energy harvesting, and catalytic domains.

However, it is important to note that the study of plasmon-generated currents is still in its early stages.^22−24^ Especially when looking at large-area nanostructured materials with significant gaps between the nanostructures, there is a lot we have yet to discover, and efforts are needed to better understand the complex physics of these nanomaterial systems. Within this context, we showcase the anomalous effects of an oscillating optical field achieved by gradually switching the light on and off at low frequencies (1, 2, 4, 6, and 8 Hz) on a large-area LSPR substrate. Our findings demonstrate the successful transformation of large-gapped cascaded nanoplasmonic structures into a state of significantly enhanced polarizability. This transformation enables the generation of light-induced oscillating electrical currents, which can be effectively modeled through an electrical circuit. Our observed signatures of current suggest that the conductivity of large-gapped nanostructure surfaces can also be tuned with light.

Concurrent electrical (current) and optical (ultraviolet–visible (UV–vis) spectroscopy) measurements were obtained using the setup illustrated in Figure 1a. The setup consisted of a light-emitting diode (LED) controlled by a sine wave function from a function generator. The electrical and optical responses were acquired using a femto/picoammeter and a spectrophotometer, respectively (details are given in the methods section within the Supporting Information). Please note that all instruments (ammeter, spectrophotometer, function generator, etc.) that were used for trigger and signal acquisition were completely isolated from the optical path of the substrate, as the LSPR substrate was optically and electrically shielded; details of the setup are shared in the Supporting Information. The LSPR substrate used in this study, displayed in Figure 1b, consisted of gold nanoparticles (AuNPs) with sizes ranging between 5 and 100 nm that were separated by distances ranging from 25 to 100 nm and had an aspect ratio of 1.65 (Figure 1c; more details are in the Supporting Information). Finite element simulation results, depicted in Figure 1d, show that the oscillations of free electrons associated with LSPR created an electric field around the nanostructures. We varied the nanoparticle size and shape in the simulation to account for morphological variability in our large-gapped nanoplasmonic/LSPR substrate consisting of AuNPs. The simulation revealed an electric field (*E*) ranging from 10 to 90 μV/m between the nanoparticles, which can explained with eq 1:
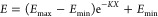
1where *E*_max_ is the maximum electric field, *E*_min_ is the minimum electric field, *K* is rate of electric field decay, and *X* is the interparticle distance (see the fitted trend in Figure 1e). With these simulations, we aimed to demonstrate the existence and enhancement of the electric field around the NPs at the interparticle distances observed in the study. Notably, the electric fields of one NP influenced the electric fields of the surrounding NPs, resulting in field enhancement currents, typically in the femtoampere (fA) range. Measuring fA-scale photocurrents from nanostructures can be challenging, as it is nontrivial to isolate unwanted noise and dark currents from the measurement system. However, we found that modulating the light intensity at a low frequency (ranging from 1 to 8 Hz) can help exclude such noises and enable the detection of low currents. Such light modulation leads to oscillating currents that increase and decrease during the on/off bursts of light, mimicking the rhythmic behavior of human breathing (Figure 1f), and therefore, we term this current as breathing current.

**Figure 1:**
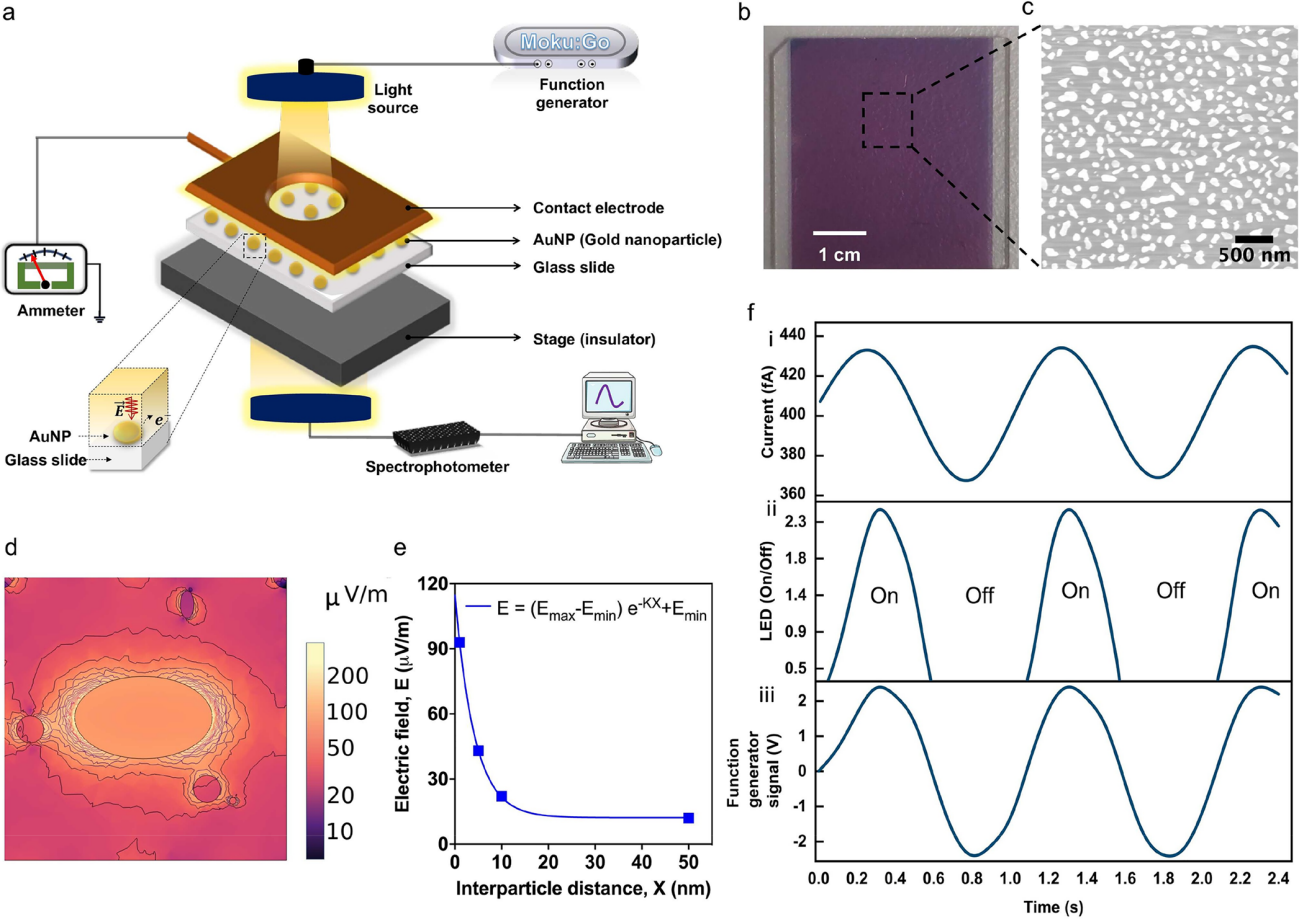
Measurement scheme and characterization features of nanoplasmonic substrate. (a) Scheme for concurrent electrical and optical measurements, consisting of a light source controlled by a function generator, ammeter, and spectrophotometer connected to a computer for measurement display. The setup also shows a glass slide consisting of the gold nanoparticle (AuNP) substrate placed on an insulating stage and the top contact electrode, through which light-induced currents are measured. (b) Picture of a glass slide consisting of AuNP. (c) Scanning electron microscopy (SEM) image showing the morphological features of the gold nanoparticles (AuNPs). This image was acquired at 20 000× (magnification) with a 10 kV accelerating voltage. d) Simulation plot showing the electric field between nanoparticles of different shapes and sizes (as seen from SEM). (e) Simulation plot demonstrating how the electric field decays when the distance between AuNPs is increased. Note that the simulation in (d) and (e) was performed using COMSOL 6. (f) A representation of breathing current alongside the light modulation (on/off) obtained by controlling the LED using a sine wave (here, frequency of 1 Hz) through the function generator.

The obtained electrical response of the LSPR substrate, including its breathing current characteristics, is displayed in Figure 2. Initially, we investigated the effect of light on the glass substrate without AuNPs (Figure 2a). Both “dark” (light off condition) and “light” (light on condition) current measurements were obtained in real-time. There was no noticeable change in the dark and light conditions obtained from the glass slide compared to those obtained from the AuNPs on the glass substrate. The absolute dark current that was measured from the bare glass substrate was found to vary between 620 and 625 fA, while that measured from the AuNPs on the glass substrate was 415 fA. The decrease in current is attributed to added impedance from the nanoparticles, as observed in our impedance measurement experiments using a vector network analyzer; details are provided in the Supporting Information. We also consider this impedance in our electrical model, which is explained later. To further evaluate the observed difference between the dark and light currents for the glass and AuNPs on the glass substrate, a statistical analysis was also conducted (Figure 2b). A large number of measurements (*n* = 103) were analyzed using Šídák’s multiple comparisons test with a significance value (alpha) of 0.05. The test revealed a significant difference level of three, which is represented by the number of stars, indicating the statistical significance of the results.

**Figure 2:**
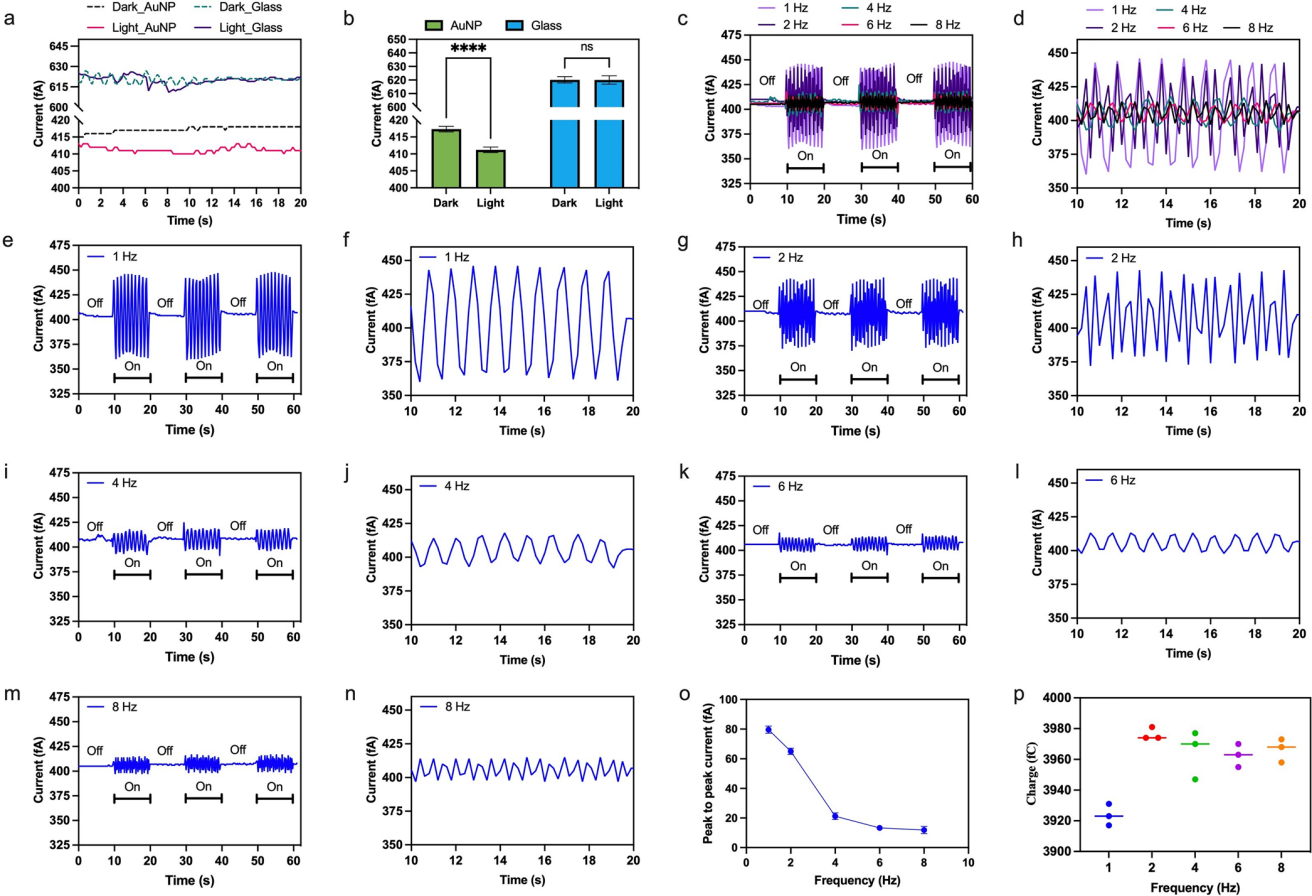
Breathing current characteristics. (a) “Dark” (light off condition) and “light” (light on condition) current measurement of bare glass substrate (as control) and AuNP on glass substrate. (b) Difference in the measured current in dark and light conditions, obtained in the experiments performed in (a). The error bars show the number of measurements (*n* = 103) conducted to obtain the data for the significance analysis test. The significance analysis was conducted using Šídák’s multiple comparisons test, where alpha (i.e., the significance value) is 0.05. The number of stars indicates the level of significance. (c) The breathing current measured when the light irradiance was modulated by a sine wave function at 1, 2, 4, 6, and 8 Hz. The plot shows the current during on/off conditions of the light source. Each on condition is called a burst. (d) The current measured during each burst when the light irradiance was modulated by a sine wave function at 1, 2, 4, 6, and 8 Hz. The measured current and the burst characteristic are also plotted individually at 1 Hz in (e) and (f), 2 Hz in (g) and (h), 4 Hz in (i) and (j), 6 Hz in (k) and (l), and 8 Hz in (m) and (n). (o) The peak-to-peak current in each burst at 1, 2, 4, 6, and 8 Hz for 10 measurements (*n* = 10). (p) The measured charge in a given burst. The charge was calculated by integrating the area under the current versus time curve. The charge measurement corresponds to three bursts. Please note that each burst was 10 s.

To accurately measure the LSPR substrate’s response to light, we employed an irradiance modulation technique by toggling the LED on and off at specific frequencies. This modulation was achieved by applying a sine wave voltage (5 V peak-to-peak) to the LED, generating frequencies of 1, 2, 4, 6, and 8 Hz using a function generator. The frequency controlled LED on/off cycles, known as bursts, were performed for a duration of 10 s (Figure 2c). During a burst, we see that the current changes for each frequency. Additionally, we observed distinct changes in the current for each frequency. Figure 2d displays the current measurements obtained during each burst for all of the frequencies mentioned above.

For each specific frequency (1, 2, 4, 6, and 8 Hz), we plotted the current measurement data individually to analyze the response characteristics. At 1 Hz, the current measurement and the corresponding burst characteristics are displayed in panels e and f of Figure 2, respectively. Similarly, at 2, 4, 6, and 8 Hz the current measurement and burst characteristics are shown in Figure 2g,i,k,m and Figure 2h,j,l,n, respectively. These plots provide insight into the impact of the LED irradiance modulation on the current. Therefore, we plotted the peak-to-peak current individually for each frequency to gain a comprehensive understanding of how the LSPR substrate responded to light irradiance modulation at different rates. From Figure 2o, we see that the peak-to-peak current decreases with an increase in the light irradiance modulation frequency. We attribute this to the capacitive behavior of AuNPs when driven by light.^25,26^ Upon increasing the frequency of the light irradiance, the AuNPs are not able to respond to fast changes in the light intensity. As a result, the peak-to-peak current decreases from 80 to 20 fA when the light irradiance frequency is changed from 1 to 8 Hz. Furthermore, integrating the burst response (current versus time plot) can provide insight on the charge changes on the nanoparticles when the light irradiance frequency is modulated (see Figure 2p).

Our experimental observations can be effectively modeled using a simple electrical circuit composed of four parts, each represented by an impedance value: *Z*_1_, *Z*_2_, *Z*_3_, and *Z*_4_ in Figure 3a–c. The LED is modeled as a diode triggered with an alternating current (AC) signal of 5 V peak-to-peak at 1, 2, 4, 6, and 8 Hz. This AC signal presents the function generation. After the LED is triggered, the light passes through air, which is represented by *Z*_3_, constituting an RLC circuit with *R*_3_, *L*_3_, and *C*_3_ as its resistive, inductive, and capacitive elements, respectively. The value of *Z*_3_ can vary according to the properties of the surrounding air, such as the temperature and humidity. Now, we move to the next impedance, *Z*_1_, which corresponds to the portion of the LSPR substrate that is exposed to light. For this, we consider an RLC (*R*_1_, *L*_1_, and *C*_1_) circuit representing the air gap between a large number of AuNPs. The AuNPs are also modeled with an inductor (*L*_n1_) and a capacitor (*C*_n1_) in parallel with each other. Previous studies have described nanoparticles with both conductive and inductive components.^27,28^ However, more recent research has also considered a capacitive behavior to account for the granular nature of nanoparticles.^13,29,30^ Building upon these findings, we introduced capacitive and inductive behavior for the AuNPs. *Z*_2_ consists of AuNPs, which are not exposed to light and are underneath the electrode contact. The electrode contact is represented by *Z*_4_. Note that we model *Z*_2_ similar to *Z*_1_, as the systems represented by these two impedances are physically similar, i.e., they represent nanoparticles with an air gap between them. However, to distinguish *Z*_2_ from *Z*_1_, *Z*_2_ is represented by *R*_2_, *L*_2_, *C*_2_, *L*_n2_, and *C*_n2_. By solving this circuit, we can obtain a simple mathematical expression (eq 2) for impedance of the LSPR substrate under light, which is primarily responsible for generating the measured current in our system. In this expression, *Z*_measured_ refers to the measured impedance, which can be measured using an impedance analyzer. More detailed explanations on the circuit elements of this expansion of the equation in terms of lumped elements are shared in the Supporting Information.
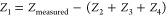
2Using numerical methods, we calculated the total current measured at the circuit’s output using an ammeter while varying the frequency. The measured current responses at frequencies 1, 2, 4, 6, and 8 Hz are shown in Figure 3d–h, respectively. These current responses exhibit oscillating behavior similar to the trends observed in the experimental measurements discussed in Figure 2. Moreover, as we link these breathing currents to the electric field enhancement resulting from plasmon formation, our study also investigates the wavelength dependence of the electric field between particles. Figure 4 shows the results of the finite element simulation, which also took into account the impedance of the nanoparticles, as explained by eq 2. Here, Figure 4a–h illustrates the electric field distribution when the nanoparticles were illuminated with light of wavelength 50, 300, 500, 555, 600, 750, 900, and 1100 nm, respectively.

**Figure 3:**
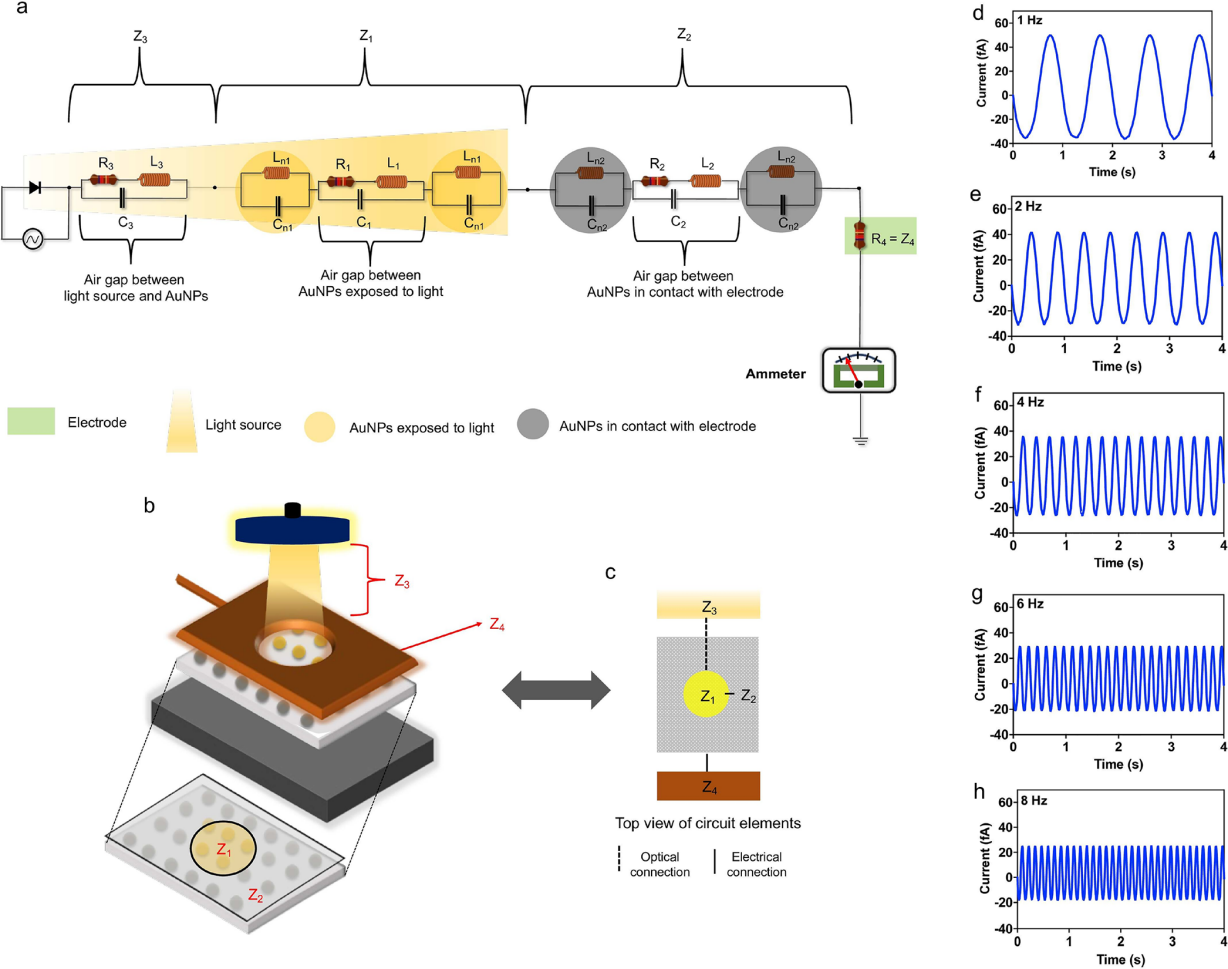
Electric analogue of breathing currents. (a) Schematic of an equivalent electrical circuit representing the overall system (including the LSPR substrate, light source, function generator, nanoparticles, the gap between them, and the metal contacts used to measure the current), developed for measurement of the breathing currents. (b, c) The location of different impedance components within the measurement system, where (c) shows a top view of the electrical connections. (d–h) The current generated from the circuit at this output (as read by an ammeter) at 1, 2, 4, 6, and 8 Hz, respectively.

**Figure 4:**
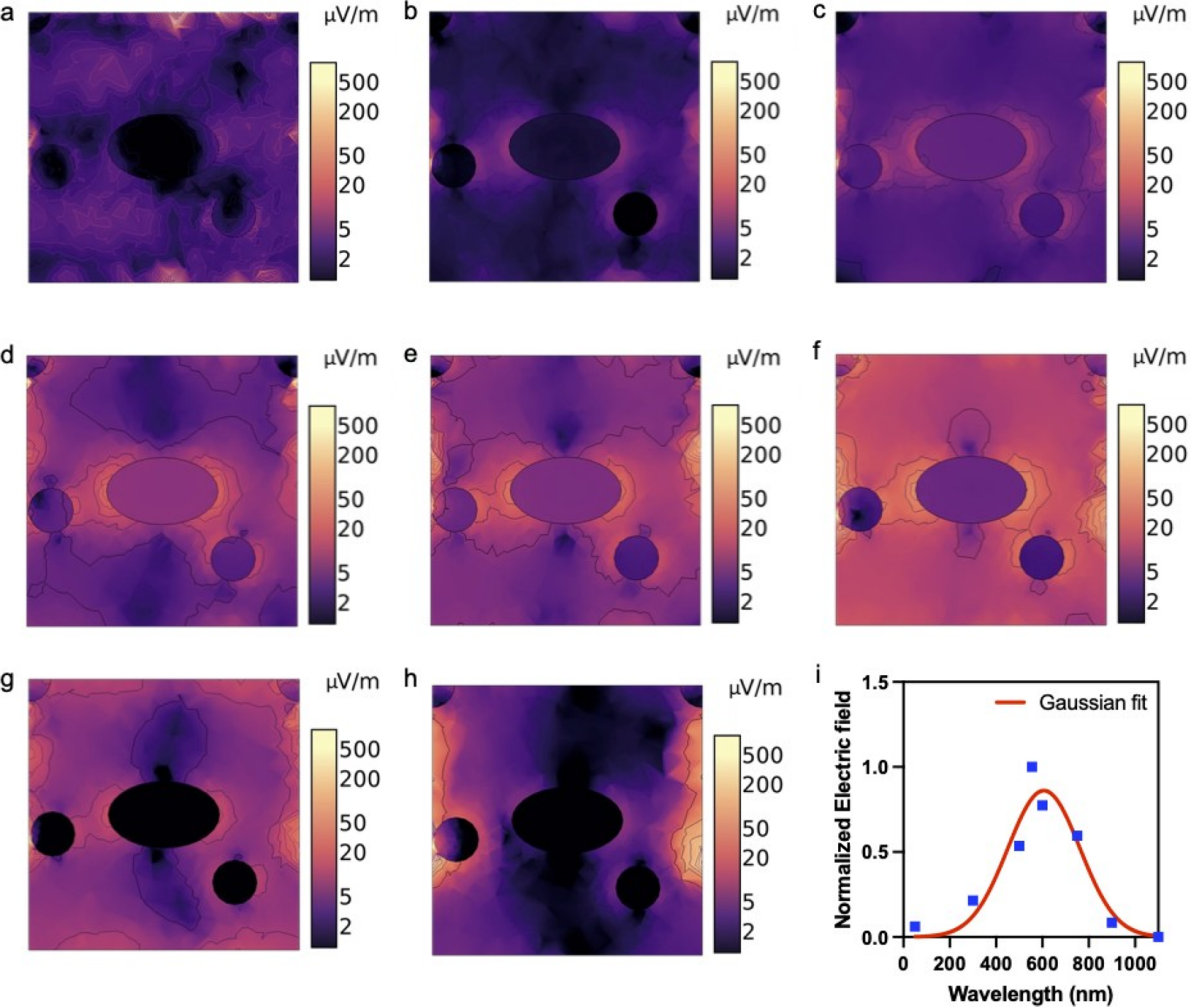
Wavelength dependency of the electric field causing the breathing currents. Visualization of the electric field distribution as nanoparticles are exposed to light at wavelengths of (a) 50, (b) 300, (c) 500, (d) 555, (e) 600, (f) 750, (g) 900, and (h) 1100 nm, showcasing the diverse responses across the spectrum. (i) Normalized electric field intensity as a function of wavelength, which is fitted with a Gaussian curve.

The normalized electric field intensity is depicted in Figure 4i, which reveals, through a Gaussian fit, that the maximum electric field intensity occurs between 550 and 600 nm, corresponding to the wavelength of the LSPR peak. Our simulations also indicate the existence of electric fields when AuNPs are exposed to wavelengths both below and above the LSPR peak wavelength. We attribute this phenomenon to the thermal excitation of the system upon interaction with light, which may also generate a current (that is lower than the plasmonic breathing current since the electric field intensity is lower than that observed at the LSPR peak wavelength). In our future studies, we aim to explore this aspect further by investigating monochromatic light sources at different wavelengths, which would require a new experimental and measurement setup. This will allow us to more distinctly separate the exclusively LSPR-driven current from the thermally excited current. However, the current focus is on illustrating the breathing current driven by the dynamic irradiance of light.

In our simulation, we also observed a reduction in the observable breathing current with higher frequencies of light irradiance, as seen in Figure 5a. This reduction in peak-to-peak current is consistent with the trend observed in our conducted experiments; also see Figure 5a. The optical response from the LSPR substrate was also recorded during the burst mode at different frequencies (Figure 5b). While a slight decrease in the peak absorbance can be observed when the frequency was changed from 1 to 8 Hz, these minute shifts are attributed to inevitable random errors, resulting from measurement to measurement, as observed in Figure 5c. Similarly, no distinguishable wavelength shifts were observed upon light irradiance modulation. Also within this figure, the induced charge (calculated by integrating the current versus time response), total absorbance (area under the UV–vis spectrum), and LSPR peak wavelength are plotted as functions of frequency. Note that the absorbance, peak wavelength, and charge values plotted in Figure 5c are normalized to compare the effect of optical changes with the measured current/charge.

**Figure 5:**
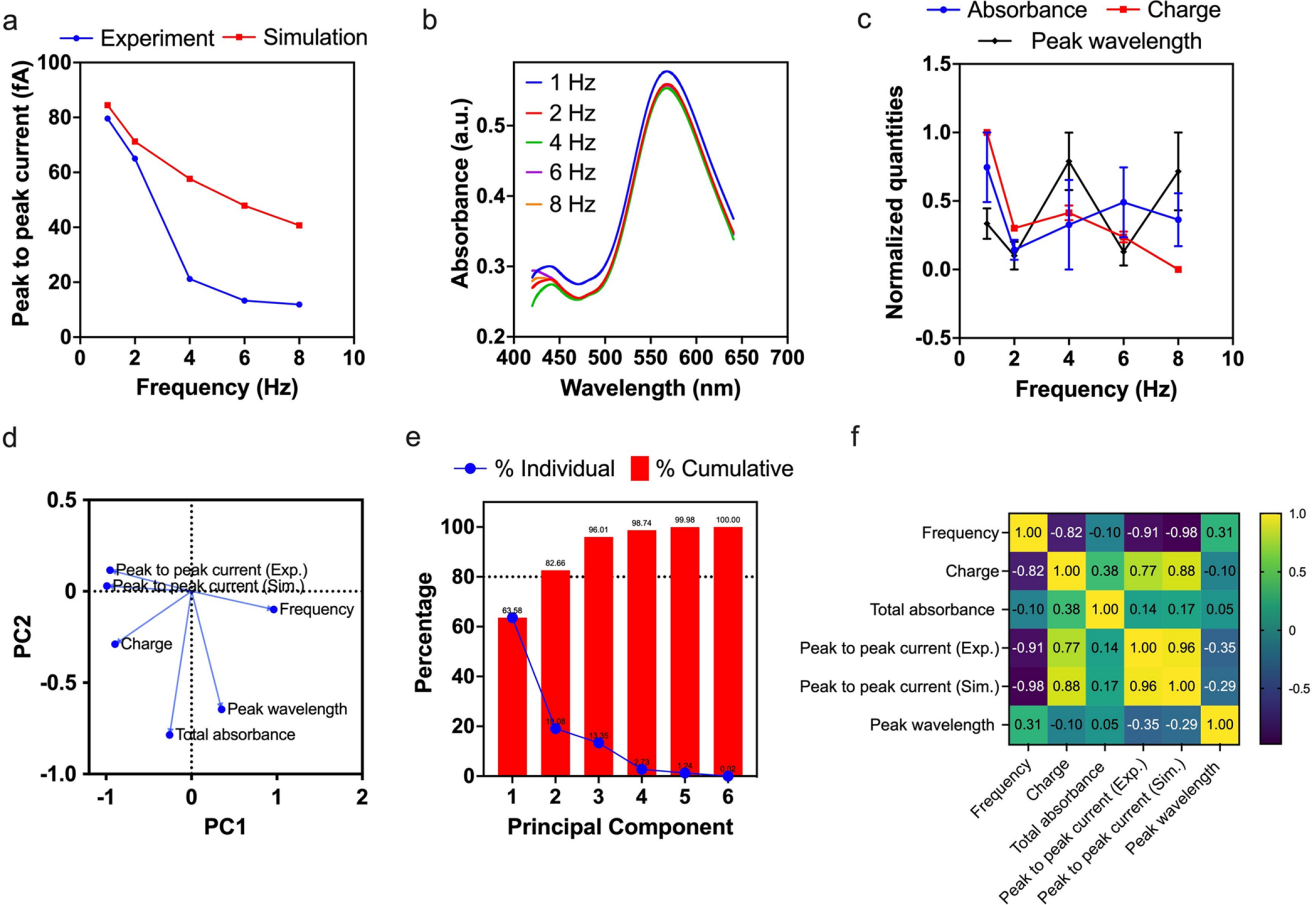
Comparison and statistical analysis. (a) Experimental and simulation peak-to-peak breathing current–frequency response. (b) UV–vis spectroscopy measuring the absorbance spectrum of the AuNPs. (c) Normalized charge, absorbance, and peak wavelength versus frequency responses. (d) Loading plot generated by principal component analysis showing the relationship between different variables measured in this work. (e) Variance among the principal components. (f) Correlation matrix depicting relationship between different variables measured in this work.

To further visualize these relationships between the experimental measurements (current, total absorbance, LSPR peak wavelength, and charge), the simulation current predicted by our analytical model, and the frequency, we performed principal component analysis (PCA). We performed PCA because it enables us to comprehend and identify significant patterns among the analyzed variables; see Figure 5d, which shows the relationships between the variables. The method for selection of the two principal components (PC1 and PC2) was based on the variance among the PCs.

As a rule, we chose the least number of components that account for at least 80% of variance in the data. Within this context, PC1 and PC2 account for a cumulative variance of 82.66% (Figure 5e). The most interesting observation from the PCA is the correlation of 0.96 between the peak-to-peak simulation current and experimental values of the same current, suggesting that the observed breathing currents can effectively be explained by our proposed analytical model; see Figure 5f. Additionally, both simulated and experimentally measured currents were found to have an inverse relationship with frequency. These currents were also found to have strong a correlation with the measured charge (0.77 and 0.88 for the experimental and simulated currents, respectively). No strong correlation was observed between the optical measurands (peak wavelength and total absorbance), suggesting that light intensity modulation had a limited influence on the LSPR peak wavelength and its absorbance.

In summary, we have presented a new approach utilizing low-frequency dynamic irradiance for quantifying light-induced currents in nanoplasmonic structures with significant gaps that surpass tunneling distances. Our study revealed that the measured current exhibited oscillatory behavior, which follows with the modulation frequency of light irradiance. Additionally, we established an electrical model that validated our experimental observations, yielding numerical results that align with the measured current. These results have the potential to significantly enhance our understanding of current dynamics within light-sensitive nanostructured materials, particularly in scenarios where nanostructures are widely spaced. The implications of these outcomes will extend to various applications within the realm of materials science and photonic systems.

## Supporting Information Available

LSPR substrate features, COMSOL simulations, experimental setup, impedance measurement of LSPR substrate and glass, electric analogue model, and materials and methods (instrumentation, electrical measurement, optical measurment, circuit modeling, LSPR substrate and morphological analysis, and simulation) (PDF)
