# Supplementary material for: Unconventional Breathing Currents Far beyond the Quantum Tunneling Distances in Large-Gapped Nanoplasmonic Systems
Source: Nano Lett. 2024 Jan 26;24(10):3157–64. doi: 10.1021/acs.nanolett.3c05133 (PMC10941250; doi:10.1021/acs.nanolett.3c05133)
Supplement: Supplementary file 1 — nl3c05133_si_001.pdf [file nl3c05133_si_001.pdf]

**Supporting information**

# Unconventional breathing currents far beyond the quantum tunneling distances in large-gapped nanoplasmonic systems

Aravind Satheesh,[†,‡] Chia-Ming Yang,[‡,¶,§,‖,⊥] Vilas Gaidhane,[#] Neeru Sood,[@] Nilesh Goel,[#] Selim Bozkurt,[†] Krishna Kumar Singh,[△] and Nikhil Bhalla[*,†,∇]

†Nanotechnology and Integrated Bioengineering Centre (NIBEC), School of Engineering, Ulster University, 2-24 York Street, Belfast, BT15 1AP, Northern Ireland, United Kingdom

‡Department of Electronic Engineering, Chang-Gung University, No.259, Wenhua 1st Rd., Guishan Dist., Taoyuan City 33302, Taiwan (R.O.C.)

¶Institute of Electro-Optical Engineering, Chang Gung University, No.259, Wenhua 1st Rd., Guishan Dist., Taoyuan City 33302, Taiwan (R.O.C.)

§Department of Neurosurgery, Chang Gung Memorial Hospital at Linko, No. 5, Fuxing St.,Guishan Dist., Taoyuan City, 33305, Taiwan (R.O.C.)

"Department of Materials Engineering, Ming-Chi University of Technology, 84 Gungjuan Rd., Taishan Dist., New Taipei City 243303, Taiwan (R.O.C.)

⊥Department of Electronics Engineering, Ming-Chi University of Technology, 84 Gungjuan Rd., Taishan Dist., New Taipei City 243303, Taiwan (R.O.C.)

#Department of Electrical and Electronics Engineering, Birla Institute of Technology and Science (BITS), Pilani Dubai Campus, Dubai International Academic City · P.O. Box: 345055, Dubai, United Arab Emirates

@Department of Biotechnology, Birla Institute of Technology and Science (BITS), Pilani Dubai Campus, Dubai International Academic City · P.O. Box: 345055, Dubai, United Arab Emirates

ODepartment of Physics, Birla Institute of Technology and Science (BITS), Pilani Dubai Campus, Dubai International Academic City P.O. Box: 345055, Dubai, United Arab Emirates

∇Healthcare Technology Hub, Ulster University, 2-24 York Street, Belfast, BT15 1AP, Northern Ireland, United Kingdom

*E-mail: n.bhalla@ulster.ac.uk

## Table of Contents


1. LSPR substrate features ........ 3
2. COMSOL simulation ........ 3
3. Experimental setup ........ 6
4. Impedance measurement of LSPR substrate and glass ........ 9
5. Electric-analogue model ........ 10
6. Materials and methods ........ 12
   6.1 Instrumentation ........ 12
   6.2 Electrical measurement ........ 13
   6.4 Circuit modeling ........ 13
   6.5 LSPR substrate and morphological analysis ........ 14
   6.6 Simulation ........ 14
7. References ........ 15

## 1. LSPR substrate features

Structural analysis for SEM image of nanoparticles distribution on glass substrate showing diameter, interparticle spacing and the aspect ratio in **Figure S1**. These attributes reveal the characteristics of LSPR chip with an average diameter of 75 nm (assuming the particles are perfect circle), and average interparticle distance 70 nm and an aspect ratio of 1.65. Aspect ratio refers to ratio of 2 cross sectional length of the particles, major axis/minor axis-where 1 denotes perfect circle.

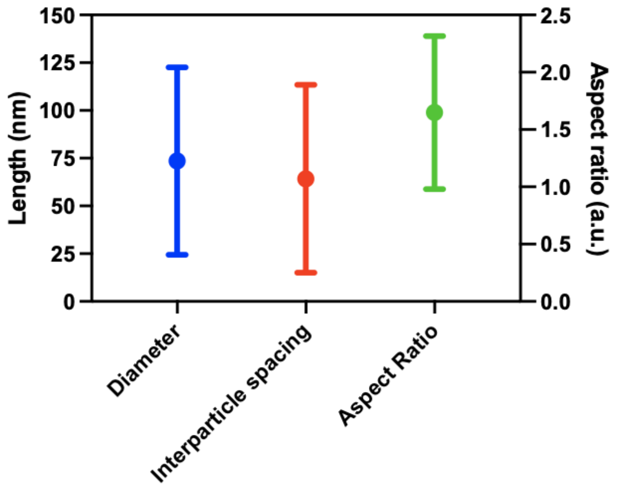


***Fig. S1.*** *Statistical analysis with diameter, spacing, and aspect ratio of Au nanostructure on LSPR chip.*

## 2. COMSOL simulation

In this section, we share the simulation studies on gold (Au) nanoparticle using COMSOL Multiphysics to primarily investigate the interaction of electric field between the nanoparticles (E). Study was conducted using "electromagnetic waves in frequency domain" module in COMSOL Multiphysics, and physical parameters used for the study are listed in table S1. Details associated with boundary conditions, for instance the direction and magnitude of the incident light that is defined by the input and output ports, in our study were incorporated from the works of Borah et. al [1]. The simulation results (**Fig. S2**) showed that as the distance between the particle increases (linearly) the electric field between the particles decreases exponentially in its value. A qualitative approach

was taken for analysing the results. Essentially, electric field at a single point was taken from the centre of the distance between the 2 nanoparticles as shown in **Fig S3**.

***Table. S1.*** *Parameters used for the simulation with their values.*

| Name | Value | Description |
|---|---|---|
| Radius | 10 nm | Radius of nanosphere |
| Interparticle distances | 1 nm, 5 nm, 10 nm, 50 nm | Distance between nanoparticle |
| Wavelength | 555 nm | Wavelength of excitation light |
| $I_o$ | 1 nW/m$^2$ | Intensity of light |

The dielectric properties for the material gold in our study were incorporated from Johnson et al. [2].

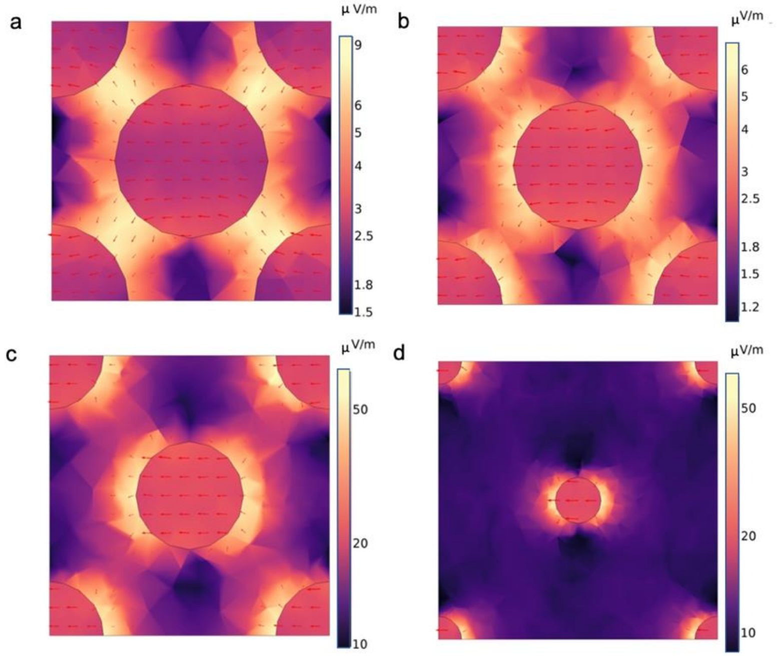


***Fig. S2.*** *Simulation of electrical field interaction between Au nanostructure for varying interparticle distances of a)1 nm, b) 5 nm, c) 10 nm, d) 50 nm.*

The values for the real and imaginary parts of gold nanoparticles were interpolated in the software for each wavelength of light used. The wavelength of the light source was set at 555 nm. Meshing is done separately for the air around the nanostructures using free triangular element type mesh and nanoparticles were meshed using tetrahedral element

type. A qualitative analysis of results from the simulation shows that the electric field between the nanoparticles decreases with increase in the interparticle distance as seen in **Fig S2**.

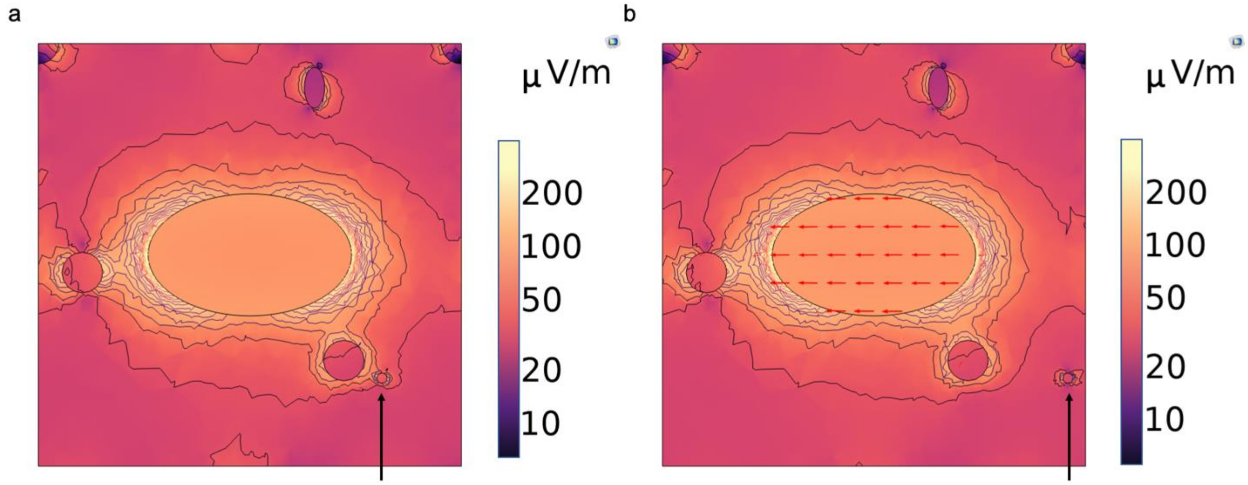


***Fig.S3.*** *Electric field interaction between particles decreases with an increase in interparticle distance, a) low interparticle distance leads to higher interaction b) higher interparticle distance leads to lower interaction.*

From the structural analysis of SEM image of the gold nanoparticle (**Fig. S1**) we observe that the interparticle distances as well as particle diameter vary between 20 nm to 125 nm. As the aspect ratio fluctuates between 1 to 2, the particles are either elliptical and quasi circular in shape. Simulation to understand the distribution of particle would rather give a closer depiction of the real interaction. Therefore, our simulation results shows that the closer particles have stronger interaction which leads to a pathway for a possible movement of electrons (**Fig. S3**). From **Fig. S3**, it is seen that the smaller nanoparticle (highlighted with an arrow) which is closer to the rest of the cluster, appears to have a strong interaction compared to the condition when it was moved away from the cluster.

## 3. Experimental setup

Details of the experimental setup are shared below in Figure S4.

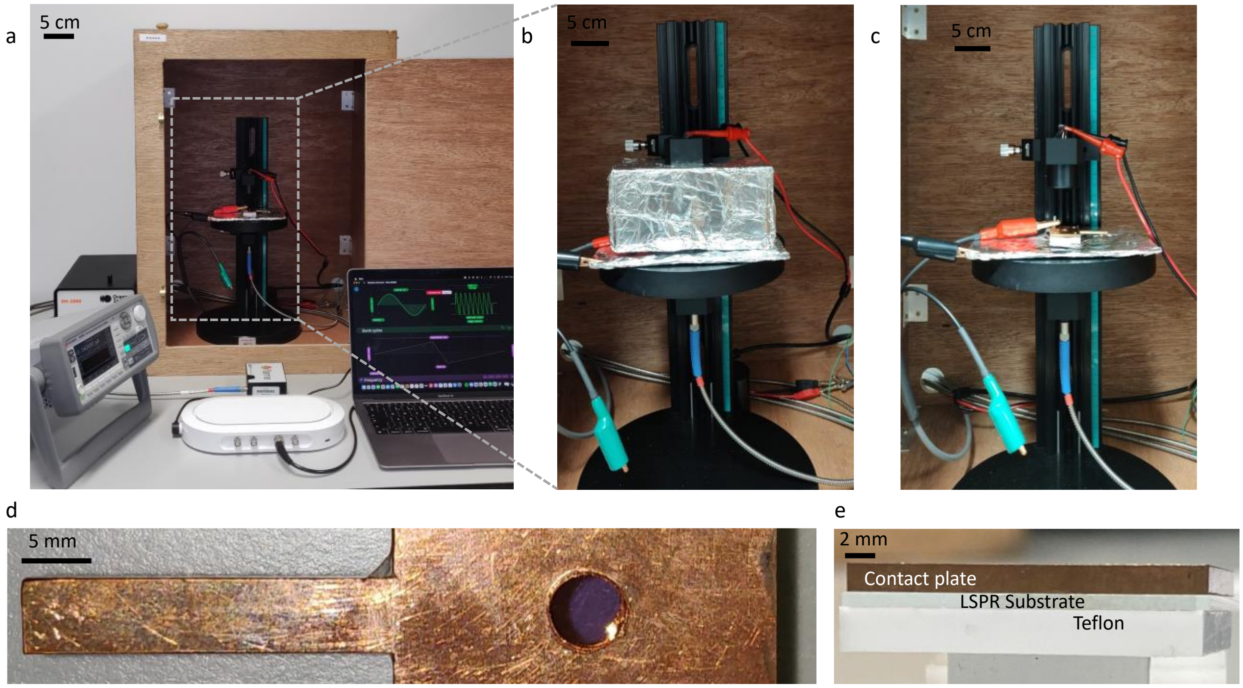


***Fig.S4.*** *Pictures of experimental setup a) depict the complete setup along with instruments used in this study; b) shows the interior part of the cage illustrating the stage on which faraday cage covers the sample.; c) shows the connections inside the faraday cage d) shows the top view of the contact plate and the central area (light pink in colour) shows the sandwiched LSPR substrate between Teflon and the contact plate. e) shows the side view of the sandwiched system shown in sub-figure d.*

The setup in **Fig. S4** shows the setup in which the experiment was conducted. Figure S4. b-c. shows the Faraday cage inside where the test stage is kept and it is also connected to a pico-ammeter for electrical measurements. The holder with a hole on the contact allows light to pass through the LSPR substrate and the side view of the holder shows sandwiched substrate insulated with Teflon and a contact plate on top.

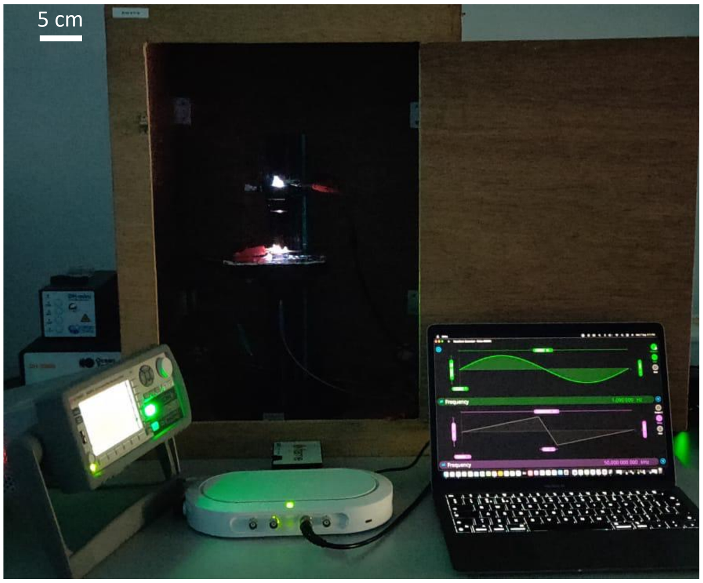


***Fig.S5.*** *The setup in dark ambient condition to eliminate the optical noise.*

Measurement set up in dark ambient conditions to eliminate optical noise from the measurement (**see Fig.S5**). It should be noted that the light is aligned directly on the LSPR substrate using the lens orifice of the stage. We have also brought the light source closer to the substrate to more focus the light in comparison to system shared in **Fig. S5**. This is achieved using a optical fibre probe (purchased from ocean insight) shown in figure **Fig. S6**.

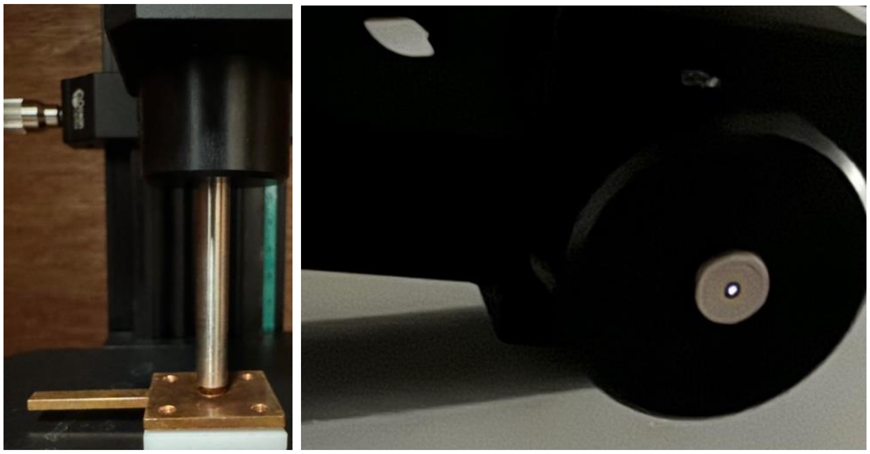

***Fig. S6.*** *Optical probe used for illumination.*

However, the probe introduces instability issues in the system as indicated by **Fig. S7**. The potential instabilities observed could stem from RF noise originating from the probe.

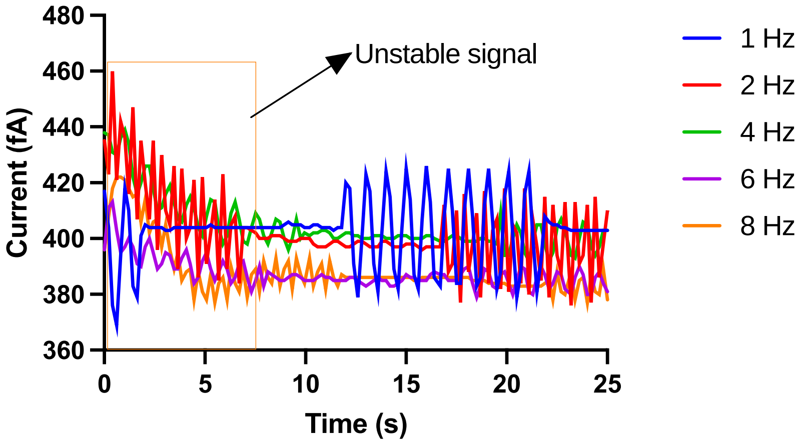


***Fig. S7.*** *Breathing current response with use of optical fibre probe used for illumination.*

The presence of this noise, leading to instability, complicates the task of accurately distinguishing the breathing current during the ON cycle at frequencies of 6 Hz (**Fig. S8**) and beyond. To address this issue, we propose employing an LED positioned at a distance from the substrate, precisely aligned with the specific area requiring light exposure.

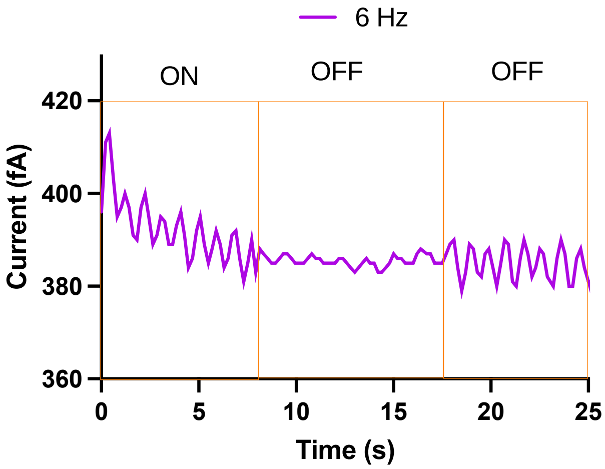


***Fig. S8.*** *Breathing current response in 6 Hz light modulation using optical probe for illumination*

## 4. Impedance measurement of LSPR substrate and glass

We measured the impedance of the LSPR substrate and the glass using a vector network analyzer (NanoVNA-H 4). We further break the measured impedance into resistance (**Fig. S9**) and reactance (**Fig. S10**) which clearly shows that adding Au on glass increase inductance (positive reactance in the inset) and capacitance (negative reactance in the inset) which is attributed to overall increase in the impedance. Moreover, with exposure to light both, resistance and reactance increase at lower frequencies which leads to decrease in the current of the gold nanoparticles substrate when exposed to light.

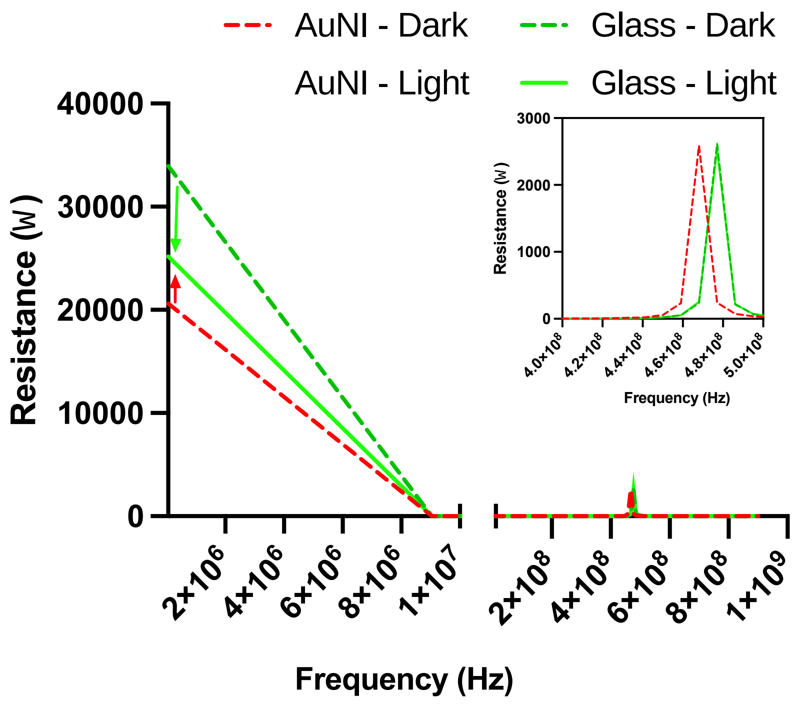


***Fig. S9.*** *Resistance of LSPR substrate*

When the glass substrate is exposed to light, a decrease is observed in both resistance and reactance. This intriguing occurrence can be attributed to the polarization effect on glass induced by the inherent impurity atoms within the borosilicate glass, signifying interaction between light and the borosilicate's atomic composition.

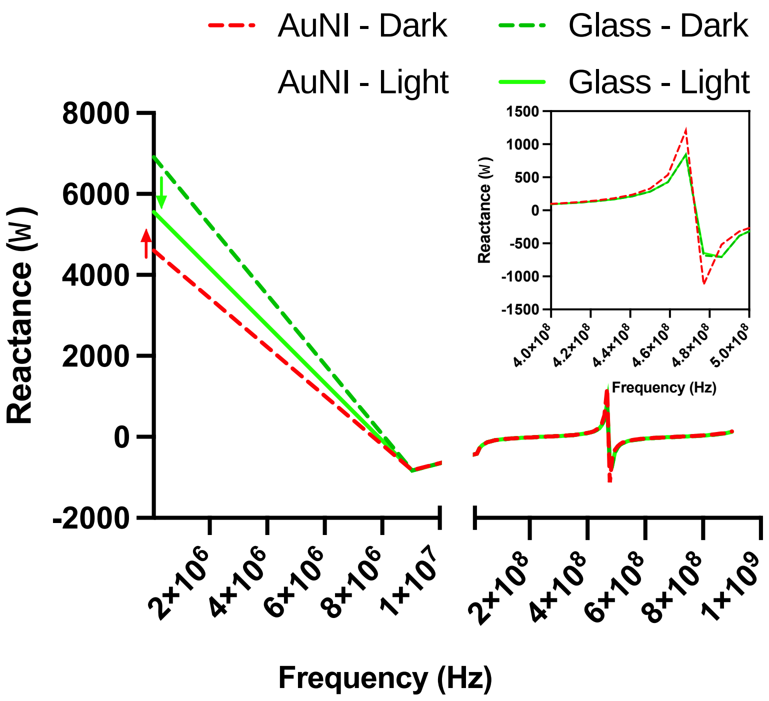


***Fig. S10.*** *Reactance of LSPR substrate*

**5. Electric-analogue model**

The circuit shown in Figure 3a within the main manuscript is also shared here (***Fig. S11***) to further explain the individual components of the model contributing to 5 different impedances considered in our proposed electric-analogue model.

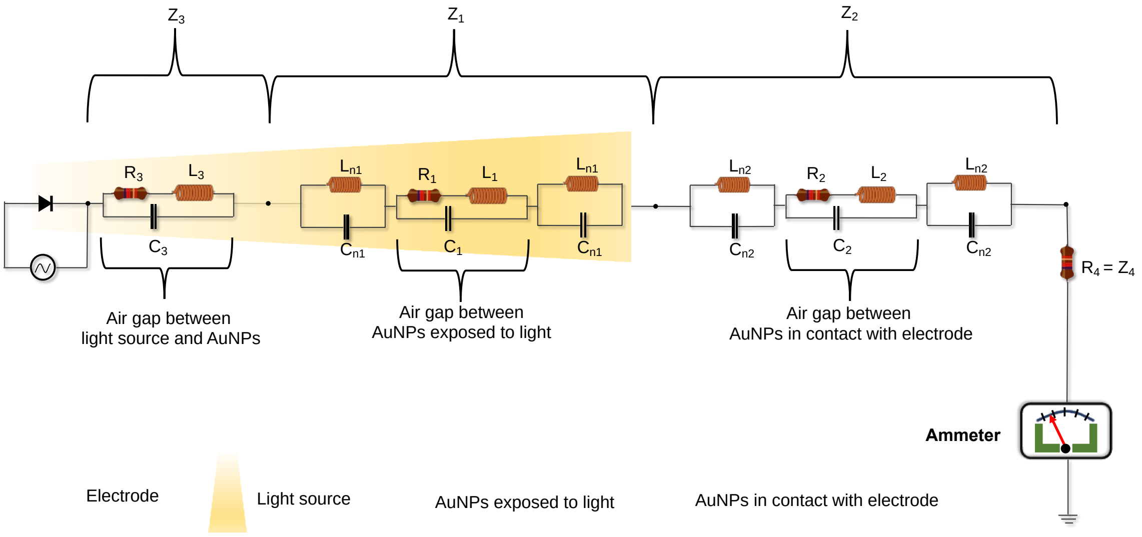


***Fig.S11.*** *Circuit representing the electric-analogue model.*

With the above circuit, $Z_1$, $Z_2$, $Z_3$, and $Z_4$, can be expressed as follows using equations S1, S2, S3 and S4 respectively:

$$Z_1 = \frac{2\,j\omega L_{n1}}{-\omega^2 L_{n1} C_{n1} + 1} + \frac{R_1 + \omega L_1 j}{\omega R_1 C_1 j - \omega^2 L_1 C_1} \tag{S1}$$

$$Z_2 = \frac{2\,j\omega L_{n2}}{-\omega^2 L_{n2} C_{n2} + 1} + \frac{R_2 + \omega L_2 j}{\omega R_2 C_2 j - \omega^2 L_2 C_2} \tag{S2}$$

$$Z_3 = \frac{R_3 + j\omega L_3}{j\omega R_3 C_3 - \omega^2 L_3 C_3} \tag{S3}$$

$$Z_4 = R_4 \tag{S4}$$

From circuit analysis we can find the total $Z_{measured}$ i.e. the measured impedance by the electrometer or impedance analyzer, given by equation S5.

$$Z_{measured} = Z_1 + Z_2 + Z_3 + Z_4 \tag{S5}$$

Adding S1, S2, S3 and S4 within S5 we get:

$$Z_{measured} = \frac{2\,j\omega L_{n1}}{-\omega^2 L_{n1} C_{n1} + 1} + \frac{R_1 + j\omega L_1}{j\omega R_1 C_1 - \omega^2 L_1 C_1} + \frac{2\,j\omega L_{n2}}{-\omega^2 C_{n2} L_{n2} + 1} + \frac{R_2 + j\omega L_2}{j\omega R_2 C_2 - \omega^2 L_2 C_2} + \frac{R_3 + j\omega L_3}{j\omega R_3 C_3 - \omega^2 L_3 C_3} + R_4 \tag{S6}$$

Equation S6 can be solved to compute the overall impedance. The equation can further be used to find the values of the individual component (resistors, inductors, and capacitors) by fitting the measured current within experiments or it can be used to find the expected current in the experiments with the help of known values of the individual electrical components. For the simulations the values used to fit are shared in **Table S2**.

***Table S2*** *: Typical values of the components used in the circuit.*

| | | **Description** |
|---|---|---|
| $R_1$ | 110 MΩ | Air between nanoparticles (under light) |
| $L_1$ | 1 mH | Air between nanoparticles (under light) |
| $C_1$ | 0.011mF | Air between nanoparticles (under light) |
| $L_{n1}$ | 100mH | Nanoparticle (under light) |
| $C_{n1}$ | 1mF | Nanoparticle (under light) |
| $R_2$ | 110 MΩ | Air between nanoparticles (under dark) |
| $L_2$ | 1 mH | Air between nanoparticles (under dark) |
| $C_2$ | 0.011mF | Air between nanoparticles (under dark) |
| $L_{n2}$ | 100mH | Nanoparticle (under dark) |
| $C_{n2}$ | 1mF | Nanoparticle (under dark) |
| $R_4$ | 100KΩ | Copper plate |
| $R_3$ | 110 MΩ | Air (under light) |
| $L_3$ | 1 mH | Air (under light) |
| $C_3$ | 0.011mF | Air (under light) |

## 6. Materials and methods

### 6.1 Instrumentation

Electrical measurements were acquired using self-calibrated Femto/Picoammeter( Keysight, B2985A). The current was captured using a cable (16494A,1.5m). The optical measurements of absorbance was measured with the help of Flame, Ocean optics (Flame-T-XR1-ES) connected with patch cords. Illumination of light source was controlled

using a function generator (Moku:go) which was connected to a white light LED ( Farnell, MP-703-1066) with a Bayonet Neill– (BNC) to crocodile probe clip cable. The entire testing was performed on a specially crafted insulated table/stage, accompanied by a homemade Faraday cage constructed from aluminum foil (Bacofoil), which was also grounded.

### 6.2 Electrical measurement

Electrical measurements were taken using Keysight B2985A which was connected to the LSPR substrate sandwiched between a teflon holder and a contact plate to capture the electrical responses from the surface of the chip. The test stage was connected to the ground to reduce the electrical noise and was placed inside a wooden enclosure to reduce the optical noise. A customized faraday cage using aluminium foil was also used to avoid electrical noise. Light illumination was controlled using LED connected to a function generator. The frequency of On and Off was controlled using the burst mode where the sine wave switching was controlled by predefined window of 10 seconds. The raw data acquired was then analyzed and plotted using graph pad prism 10.1.

### 6.3 Optical measurement

The UV-Vis LSPR spectrum was obtained using a custom experimental setup at Ulster University, UK. This setup was assembled with components sourced from Ocean Insight, including a spectrometer FLAME-T-XR1-ES, UV-Vis patch connectors, LED (Farnell, MP-703-1066), RTL-T stage, and Ocean View software. Before obtaining the LSPR spectrum, background noise cancellation was performed by measuring dark and reference signals using a glass slide as a reference. The glass slide used for reference was the same substrate on which nanoparticles were deposited. Subsequently, all the collected data was analyzed and plotted using the integrated features of GraphPad Prism 10.1 software.

### 6.4 Circuit modeling

The circuit diagram given in Figure 3 (main manuscript) was developed using the voltage source, diode, resistance, inductance, capacitance, and ground elements under specialized power systems library in the SimScape electrical in Matlab 2021b and NI

multisim simulator. Voltage and current in the circuit were measured using current and voltage measurement elements. Measured voltage and current time series were visualized using sink elements in Simulink. Input voltage in the circuit was changed between -2.5 Volts and 2.5 Volts at 1 Hz, 2 Hz, 4 Hz, 6 Hz, and 8 Hz frequencies in the simulations. The set of equations was also solved using Maple 2023 software.

### 6.5 LSPR substrate and morphological analysis

The LSPR substrate consisting of nanoparticles were purchased from NanoSPR devices, USA, product number: BA3120. The LSPR chip's morphology, was characterized using a field emission scanning electron microscope (Hitachi SU5000) in low-vacuum mode. The image was acquired at 20,000 time magnification at a 10 kV accelerating voltage. Image analysis used to uncover morphological characteristics, including particle diameter, aspect ratio, and spacing was carried out by directly using the built-in functions available within the ImageJ software.

### 6.6 Simulation

The Finite element analysis (FEA) was performed using COMSOL 6.1. The light driven characteristics for LSPR were modeled using the computational electromagnetics module. Clusters of gold nanoparticles (AuNP) of elliptical and spherical shape under light (wavelength of 555 nm) illumination with different interparticle distance were in 3-D space surrounded by air (refractive index as 1). The dielectric properties of the AuNP for each wavelength were interpolated using experimental values (details in the supporting information). The design for simulation was decided for mimicking the similar particle shape and distribution seen in the SEM analyses consisted of nanoparticles with different shape and size (with radius of 5-100 nm). The simulated results showed LSPR in AuNPs where the electrical fields were observed to merge together forming a path for electron travel. The output is presented with a qualitative measure of the electric field (in /m) formed around these AuNPs. Electric field was also observed to decrease with an increase in interparticle distance (1 nm, 10 nm, 50 nm).

## 7. References

[1] Borah, R., Ninakanti, R., Bals, S. and Verbruggen, S.W., (2022). Plasmon resonance of gold and silver nanoparticle arrays in the Kretschmann (attenuated total reflectance) vs. direct incidence configuration. *Scientific Reports*, *12*(1), p.15738.

[2] Johnson, P. B., & Christy, R. W. (1972). Optical constants of the noble metals. *Physical Review B*, *6*(12), 4370.
